# Protective Effect of Barberry (*Berberis vulgaris* L.) Extract on Oxidative Stress and Behavioral Changes in Toxicity Induced by Diazinon in the Brain Tissue of Male Rats

**DOI:** 10.1002/brb3.71710

**Published:** 2026-08-20

**Authors:** Razieh Parvareshi Hamrah, Elham Shiri, Ahmad Fadai, Fereshteh Mehri

**Affiliations:** ^1^ Research Center for Microbiology Islamic Azad University of Hamedan Hamedan Iran; ^2^ Department of Anatomical Sciences School of Medicine Hamadan University of Medical Sciences Hamadan Iran; ^3^ Nutrition Health Research Center Institute of Health Sciences and Technologies Avicenna Health Research Institute Hamadan University of Medical Sciences Hamadan Iran

**Keywords:** barberry, behavioral activities, brain, diazinon, oxidative stress

## Abstract

**Background:**

Diazinon (DZN) is a hazardous organophosphate pesticide that is applied to control insects, which causes oxidative damage in brain tissue. Barberry (*Berberis vulgaris* L.) is a plant widely used in Asia for its therapeutic properties.

**Objectives:**

The present study evaluated the protective effects of *B. vulgaris* on DZN‐induced brain damage in rats.

**Method:**

Twenty‐four male rats, aged 5–7 weeks, were divided into four equal groups of six rats; the first group (control group) received corn oil. The second group was nourished with DZN (70 mg/kg). The third group was fed with barberry extract (200 mg/kg). The fourth group received both DZN (70 mg/kg) and barberry extract (200 mg/kg). All these processes were done orally for the duration of 28 days. Oral administration of corn oil, DZN, and barberry extract was done through gavage. Biomarkers, including malondialdehyde (MDA), total oxidant status (TOS), total antioxidant capacity (TAC), and superoxide dismutase (SOD), was used to evaluate oxidative stress. After 28 days of treatment, on the morning of the 29th day, memory and behavioral activities tests were performed using the *Y*‐Maze and open field test. In addition, the hematoxylin and eosin (H&E) staining method was used for the study of histopathology.

**Results:**

As expected, DZN remarkably increased the contents of MDA and TOS and also decreased the levels of TAC and SOD in brain tissue. Data indicated that DZN induced neurotoxicity through the impairment of memory and behavioral activities. Co‐treatment with barberry extract reversed this oxidative stress, status, memory, and behavioral activities. Furthermore, barberry extract ameliorated brain histopathological injuries induced by DZN. Our findings confirmed that barberry extract had antioxidative effects against DZN toxicity.

**Conclusion:**

The present findings propose that barberry extract is a safe and promising agent for the treatment of toxicity induced by DZN.

## Graphical abstract

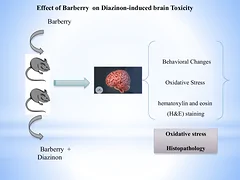

## 1 Introduction

In recent times, there has been a rise in the level of environmental contamination caused by various pollutants such as metals, pesticides, and mycotoxins on a global scale (Mehri et al. 2020). These pollutants have a significant impact on the stability of the ecosystem and the survival of living organisms (Esfahani and Mehri 2024). Pesticides, which are chemical pollutants, have detrimental effects on the environment, and their excessive usage leads to severe health issues (Esfahani et al. 2023). Diazinon (DZN), a common organophosphate (OP) pesticide, is utilized as an insecticide, acaricide, and repellent to manage insects and pests in various crops such as fruits, rice, maize, berries, grains, and mushrooms (Mehri et al. 2022). DZN (0, 0‐diethyl‐0‐[2‐isopropyl‐6‐methylpyrimidin‐yl] phosphorothionate) is widely used for controlling insects and improving agricultural productivity. Despite its effectiveness against insects, DZN has been found to have adverse effects on non‐target species such as mammals and birds. DZN, like other OPs, functions by phosphorylating the serine hydroxyl group in the substrate‐binding domain of acetylcholinesterase (AChE), thereby inhibiting the enzyme's activity and causing an accumulation of acetylcholine (J. Singh et al. 2022). The belief that organophosphorus poisons solely induce illness by blocking AChE is no longer considered accurate (Nasr 2014). There is substantial evidence indicating that secondary cellular and molecular events are implicated in sub‐chronic and chronic intoxication, with oxidative stress (OS) playing a crucial role (Abdel‐Daim 2016). OS has been identified as the primary mechanism of toxicity induced by OPs such as DZN (Lotfi et al. 2023). OS is well known to alter the transcription and translation of specific genes and proteins responsible for governing mitochondrial functions and apoptosis (J. Singh et al. 2022). Due to its direct and indirect impact on all functions of sensitive tissues, as well as its acceleration of pre‐damage pathways leading to cell apoptosis, necrosis, and other tissue injuries, OS is viewed as a promising target for therapeutic interventions. Variations in gene expression contribute to tissue damage and abnormal organ function (Ratliff, Abdulmahdi et al. 2016). Enhanced production of reactive oxygen species (ROS) and reactive nitrogen species (RNS) can lead to OS (S. Ahmadi and Khazaei 2024); however, a certain level of these compounds is essential for cell growth, proliferation, and regulation of vascular reactivity (Di Meo et al. 2016). An imbalance between free radical generation and antioxidant defense can result in tissue damage, inflammation, and the progression of diseases. DZN increases lipid peroxidation (LPO), affecting the lipids in mitochondrial and cell membranes (R. Liu and Wu 2026). This can cause changes such as reduced mitochondrial membrane potential, increased inner membrane permeability, mitochondrial enlargement, and loss of mitochondrial components, ultimately initiating apoptosis (Boussabbeh et al. 2016). Research on DZN has indicated apoptosis and DNA damage (Wang et al. 2018). Barberry is a medicinal plant that is used as a food additive all over the world (Grădinariu et al. 2024). Its various types of therapeutic and medicinal effects have been studied not only in human models but also in animal models (Stabnikova et al. 2024). Berberine (BBR) (5,6‐dihydro‐9,10 dimethoxybenzo [g]‐1,3‐benzodioxolo [5,6‐a] quinolozinium) is one such natural alkaloid found in various medicinal herbs including *Berberis vulgaris*, *Berberis aristata, Berberis lyceum*, *Chelidonium majus*, and so on (Phogat, Singh, et al. 2023). It can account for the significant antioxidant, antimicrobial, and anti‐cancer properties exhibited by this plant. BBR is a natural alkaloid widely used as an effective therapeutic molecule owing to its antioxidant, anti‐inflammatory, anti‐apoptotic, and modulatory activities (Phogat, Singh, et al. 2023).

Extracts or individual compounds of this plant have been reported to have antiarrhythmic, antibacterial, anticholinergic, antihistaminic, hypotensive, anti‐inflammatory, analgesic, and vasodilator effects. Extracts or individual compounds of this plant have been reported. Medicinal plants help to significantly improve the level of antioxidants in the human body. Protection of some plant agents against DZN‐induced toxicity in mice has already been demonstrated (Al‐Attar 2015). Plants produce diverse combinations of compounds, which are known as antioxidants, to react against environmental stress. Natural antioxidants can reduce harmful agents, capture free radicals, act as chelating agents for pro‐oxidant metals, and suppress the formation of singlet oxygen (Muhammad 2013). Many studies have reported that the antioxidant properties of barberry extract reduce the toxicity of various poisons by reducing brain injury‐induced damage in various experimental animals (Brunetti et al. 2009). The present study aimed to investigate the protective effects of barberry extract against OS and behavioral changes induced by DZN in the brain tissue of male rats.

## 2 Materials and Methods

### 2.1 Material

DZN (Merck Co., 99% purity, Code: 075801) was obtained from the Agricultural Research, Education and Development Organization (AREDO) (Tehran, Iran). A dose of 70 mg/kg of DZN was selected based on the pilot study conducted in previous studies, equal to 1/5 of its LD50 (350 mg/kg) and was found to be the optimum dose and was administered orally to rats for 4 weeks, with or without 200 mg/kg of *B. vulgaris* extract (Mehri et al. 2022).

### 2.2 Animals

This experimental research was conducted on 24 male rats aged 5–7 weeks, which were obtained from the Laboratory Animal Research Center of the Medical University of Hamedan. These rats had a normal weight of 180–250 g. Before beginning the experiment, we kept them in cages for 1 week to adjust to the new environment at a temperature of 22°C, relative humidity of 40%, with appropriate ventilation, and 12:12 light/dark hours. We nourished them with standard specific food and tap water ad libitum in the laboratory. This study was carried out using laboratory animals and following the rules of the Medical Research Center of the Medical University of Hamedan, Iran. All experimental protocols of this study were approved under the ethical number IR.UMSHA.REC.1403.236.

### 2.3 *B. vulgaris* Extract Preparation

To prepare ethanol extracts, 50 g of ground barberry fruits were obtained from the “Scientific Collection of Living Plants” of the Botanical Garden of Qayinat in South Khorasan province. After the samples were mixed with 500 mL 96.00% ethanol, the solutions were stirred at room temperature overnight. The extracts were filtered using No. 42 Whatman filter paper (Whatman, Pleasanton, USA) and concentrated under vacuum at 45.00°C using a Heidolph rotary evaporator (Laborata 4003; Schwabach, Germany). Finally, the extracts were dried in a desiccator and kept in the dark at 4.00°C until analysis (Bursal and Köksal 2011).

### 2.4 Experimental Protocol

After deciding the effective doses, we randomly divided the male rats into four groups of six rats. The first group (control group) consisted of rats that received corn oil. The second group was nourished with DZN (70 mg/kg). The third group was fed with barberry extract (200 mg/kg). The fourth group received both DZN (70 mg/kg) and barberry extract (200 mg/kg). All these processes were done orally for the duration of 28 days. Oral administration of corn oil, DZN (solvent in corn oil), and barberry extract was done through gavage. After 28 days of treatment (to form a subacute toxicity model), on the morning of the 29th day, memory and behavioral tests were performed on all rats. Then, following the ethical principles of animal care we anesthetized the rats by intraperitoneal injection of xylazine (dose 5–10 mg/kg) and ketamine (dose 100 mg/kg). We quickly collected blood samples from the heart. In addition, we isolated the brain tissue and homogenized it in phosphate buffer, keeping it in a freezer at −80°C for biochemical experiments.

### 2.5 OS Biomarkers

#### 2.5.1 Total Protein Assay

The Bradford method was used for the assay of protein concentrations in homogenized brain tissue, and bovine serum albumin was applied as a standard (Esfahani et al. 2023).

#### 2.5.2 The Evaluation of AChE Activity

Acetylcholine enzyme activity was assessed by the method of Ellman et al. 1961 According to this technique, cholinesterase enzyme can hydrolyze 5,5′‐dithiobis‐2‐nitrobenzoic acid (DTNB) chromogen (as Ellman's reagent). A growth of absorbance at 412 nm is documented as enzyme activity. The outcomes were stated as units per liter (U/L) (Foyer and Fletcher 2001).

#### 2.5.3 Lipid Peroxidation Assay

OS causes LPO. The most significant product that was produced by the Yagi method and with spectrofluorimetry is malondialdehyde (MDA). This assay was performed with some modifications according to our previous studies. The absorbance measurement at 532 nm indicated the MDA level. The results were calculated as nmol/mg of protein (Lotfi et al. 2023).

#### 2.5.4 Measurement of Total Antioxidant Capacity, Superoxide Dismutase, and Total Oxidant Status

The ferric reducing antioxidant power (FRAP) method was used to measure total antioxidant capacity (TAC). At low pH, the Fe (III)/tripyridyltriazine complex is converted to the ferrous (Fe (II)) form, which has a bluish color. This change can be assessed by analyzing differences at the wavelength of 593 nm (Y. Liu et al. 2024). The measurement of superoxide dismutase (SOD) and total oxidant status (TOS) was performed using relevant kits and according to the kit protocol (Johansson and Borg 1988).

### 2.6 Open Field Test

The open field test (OFT) was performed in cages made of transparent Plexiglas (40 × 40 × 40 cm) as in a previous study. After oral administration of DZN for 28 days, on Day 29, rats were transferred to behavioral lab and located in the new cage. Formerly, the camera was placed above the cage, and the movement of the rats was documented for 10 min. Behavioral assessment, total distance moved, peripheral and central zone spent time for each animal were recorded using video tracking software EthoVision VR XT (Version 8, Noldus, Netherlands), and the records were analyzed for statistical analysis (Johansson and Borg 1988).

### 2.7 *Y*‐Maze Test

In this apparatus, which has three arms and is formed just like the letter Y, short‐term spatial memory and the speed of its stabilization handle are checked. The creature is set within the central portion of the maze and is permitted to move freely within the arms of the maze. Rats actually would rather investigate new places, and when they enter the central portion from one arm, they tend to enter the arms that they have not looked at recently. This phenomenon is called spontaneous alternation. If the animal`s short‐term memory is harmed, the amount of alternation will diminish significantly. The percentage of alternation was calculated for the rats that received the carrier and for the treated rats, then analyzed by the software (Johansson and Borg 1988).

### 2.8 Histopathological Studies

The procedure for sample collection involved the removal of the heads of the rats following anesthesia with ketamine and xylazine. Subsequently, the skulls were opened, allowing for the complete extraction of the brains. After being rinsed with normal saline, the brains were immersed in a 10% formalin fixative. Following dehydration and clarification processes, paraffin blocks were created from the samples. Tissue sections measuring 5 µm were sliced from the brain, and the slides underwent staining with hematoxylin and eosin. The CA1 region of the hippocampus was analyzed under a light microscope at a magnification of 400×, with 15 distinct fields of view examined for each group.

### 2.9 Statistical Analysis

The analysis of the data was conducted using GraphPad Prism software (version 5, GraphPad Software Inc.). Results are expressed as mean ± SE. The assays were carried out in triplicate, and the means were utilized for statistical evaluation. The normality of the data was initially assessed using the K–S test, and if the data met the criteria for normality, a one‐way analysis of variance was employed as needed to determine differences. A post hoc Bonferroni test was subsequently applied to assess the differences among the groups. A *p*‐value of less than 0.05 was deemed significant.

## 3 Results

### 3.1 The Effect of Barberry on AChE Activity

**FIGURE 1:**
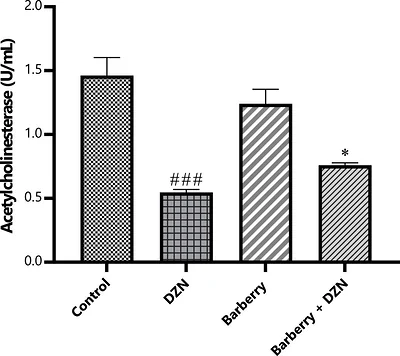
The effect of barberry on acetylcholinesterase activity. Data are expressed as mean ± SEM. ^###^ *p* < 0.001 compared to the control group, ^*^ *p* < 0.05 compared to diazinon group (*n* = 6).

The DZN group exhibited a decrease in AChE activity when compared to the control group (*p* < 0.001). Treatment with barberry extract resulted in a notable rise in AChE levels when compared to DZN (*p* < 0.05) (Figure 1).

### 3.2 Measurements of OS Biomarker

#### 3.2.1 Effect of Barberry on the Brain MDA and TOS Levels

**FIGURE 2:**
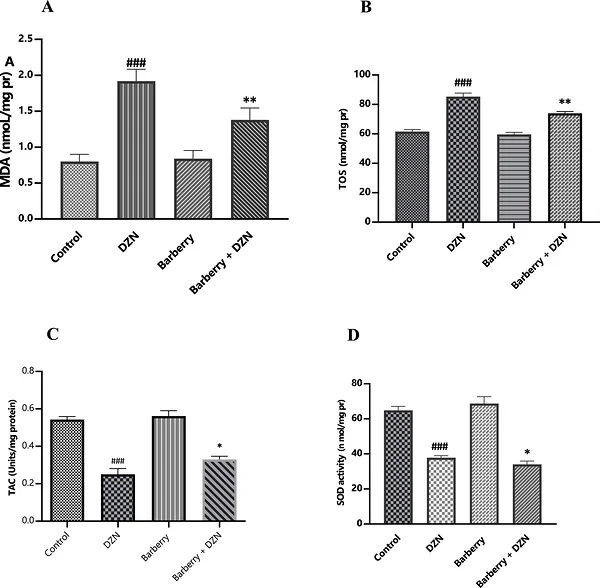
Effects of barberry on brain MDA (A), TOS (B), TAC (C), and SOD (D) levels in samples of poisoned rats by diazinon. The data are expressed as mean ± SEM (*n* = 6). ###*p* < 0.001 compared to the control group, *p < 0.05 and **p < 0.01 compared to diazinon group.

According to the results, the levels of MDA and TOS in the brain sample were significantly increased in the DZN group compared to the control group (*p* < 0.001 and *p* < 0.001, respectively). However, in the DZN group and treated with barberry extract, MDA and TOS levels in the brain were reduced to the DZN group (*p* < 0.01 and *p* < 0.01, respectively) (Figure 2A,B).

#### 3.2.2 Effect of Barberry on the Brain TAC and SOD Levels

The levels of TAC and SOD in the brain sample were significantly decreased in the DZN group compared to the control group (*p* < 0.01 and *p* < 0.001, respectively). In this regard, administration of barberry extract under DZN treatment increased brain TAC and SOD levels compared to the DZN group (*p* < 0.05 and *p* < 0.05, respectively) (Figure 2C,D).

### 3.3 Measurements of Behavioral Tests

#### 3.3.1 Open‐Field Test

**FIGURE 3:**
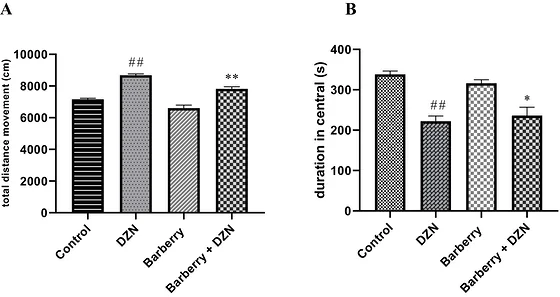
Effects of barberry on the total distance moved (A) and decreased duration in central zone (B) in samples of poisoned rats by diazinon. The data are expressed as mean ± SEM (*n* = 6). ##*p* < 0.01 compared to control group, *p < 0.05 and **p < 0.01 compared to diazinon group.

According to Figure 3A,B, 28‐day treatment with DZN significantly increased the total distance moved and decreased the duration in the central group than the control group (*p* < 0.01 and *p* < 0.01, respectively). In animals that received barberry extract and DZN, the DZN group significantly decreased the total distance moved and increased duration in the central area compared to the control group (*p* < 0.01 and *p* < 0.05, respectively).

#### 3.3.2 *Y*‐Maze Test

**FIGURE 4:**
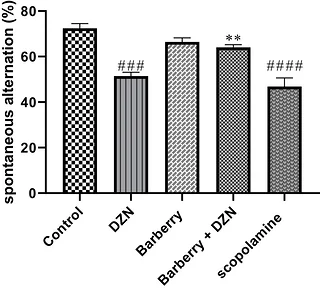
Effects of barberry on the brain passive avoidance levels in samples of poisoned rats by diazinon. The data are expressed as mean ± SEM (*n* = 6). ###*p* < 0.001 and ####*p* < 0.0001 compared to control group, **p < 0.01 compared to diazinon group.

The passive avoidance test is believed to evaluate long‐term memory in animals. Figure 4 shows that the avoidance latency of the barberry extract‐exposed animals is significantly lower than that of the control mice (*p* < 0.001), while treatment with barberry extract and DZN reduced avoidance latency compared to the control group (*p* < 0.01).

### 3.4 Histological Results

**FIGURE 5:**
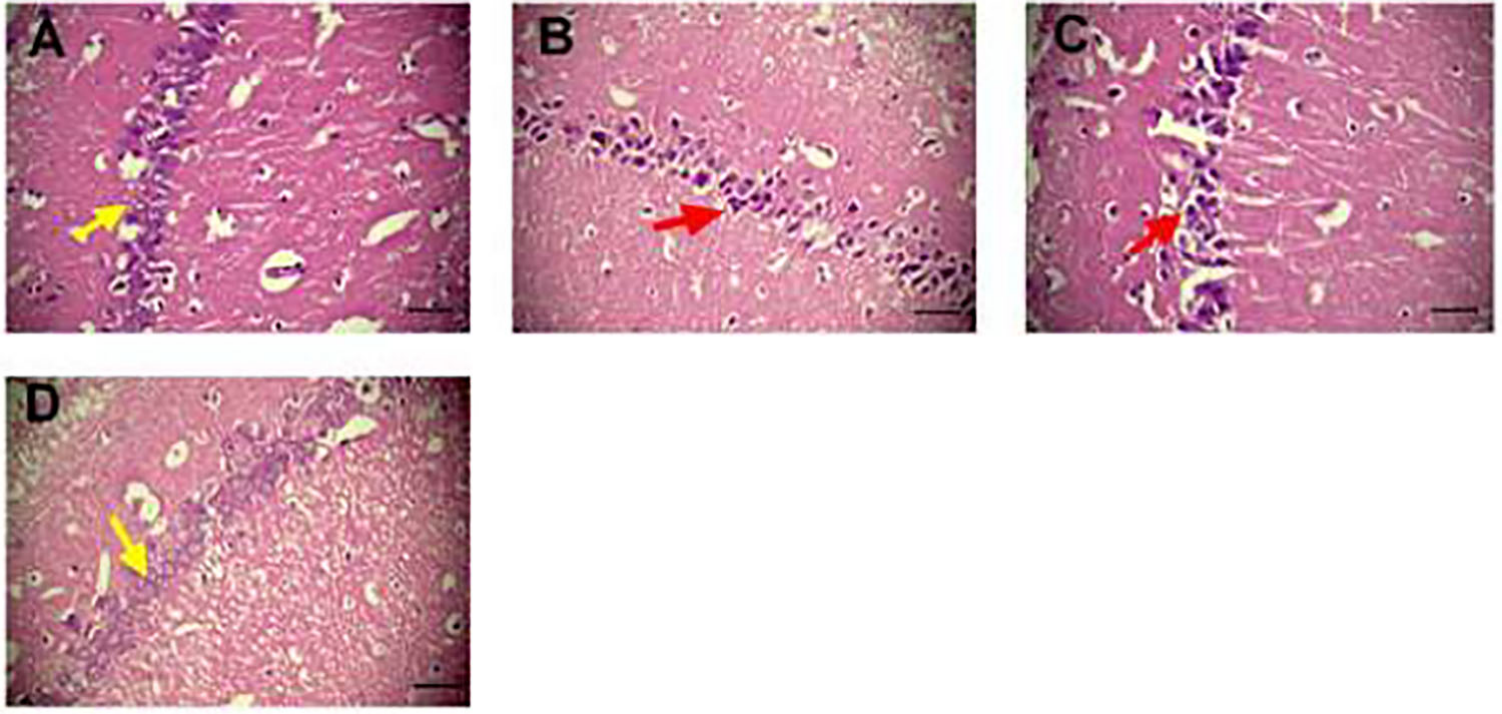
Effect of barberry on diazinon‐induced brain histopathological changes. Control group (A), barberry (B), diazinon (C), and diazinon + barberry (D); 20× magnification. The yellow arrow indicates healthy pyramidal cells and the red arrow indicates damaged cells.

Investigations showed that in the control group (Figure 5A), neurons of the pyramidal layer of the hippocampus had large central nuclei, one or more specific nuclei, and a peripheral distribution of Nissl granules. In the barberry‐receiving group (Figure 5B), a number of pyramidal layer neurons showed degenerative changes compared to the control group. These changes can indicate the toxicity of this dose because a lower dose had a better healing effect on the condition of neurons. Administration of DZN caused a significant decrease in neurons of the pyramidal layer in the hippocampus in general. The nuclei of the cells were compressed and pyknosis, which indicates the destruction of these cells (Figure 5C). Administration of barberry extract in groups receiving DZN showed the effectiveness of this extract in improving tissue conditions in terms of morphology and the number of neurons in the pyramidal layer of the hippocampus. The reduction of degenerative changes is clearly seen in this groups (Figure 5D).

## 4 Discussion

OPs have been extensively utilized as insecticides and are frequently employed in agriculture to safeguard plants from pests (Yang et al. 2024). However, the improper use of these chemicals can have detrimental impacts on human health, specifically in terms of developmental neurotoxicity (Ádám et al. 2024). Pesticides can also trigger cancer through OS mechanisms (Asadikaram et al. 2024; Y. Liu et al. 2024). DZN, part of the OPs group, is a thiophosphoric acid ester that poses ecological and biological risks in agriculture (Jeon et al. 2024). DZN induces OS and DNA fragmentation, and inhibits AChE in the brain, a membrane‐bound enzyme with lipid dependence in the brain (Delavar et al. 2024).

OS occurs when there is an imbalance between ROS and antioxidant defenses in the body (Hu et al. 2024). Studies have linked OS to neurodegenerative disorders, heart disease, diabetes, and other health conditions (Hayes et al. 2020). ROS can come from internal sources like mitochondria, as well as external sources like UV light and pollutants (Aranda‐Rivera et al. 2022). While ROS plays a role in various bodily processes, excessive amounts can contribute to chronic diseases (Kishi et al. 2024). Antioxidants protect against the harmful effects of ROS, preventing LPO and the development of chronic illnesses. It is important to maintain a balance of ROS to support overall health and well‐being (Gulcin 2020). Growing evidence from various studies indicates that DZN acts by the mechanism of OS and mitochondrial dysfunction (Shakouri et al. 2021; Dini et al. 2024).

The current investigation demonstrated that DZN exposure causes notable alterations to the antioxidant system and leads to oxidative harm in the cerebellum and cerebrum of rats. Our findings indicate that DZN reduces brain AChE activity in rats. The findings of the present study were consistent with the study of Sonei et al. (2020), who showed that AChE activity was inhibited by exposure to this pesticide.

Herbal supplements are utilized for the treatment of various ailments, and individuals nowadays exhibit a greater preference for complementary and alternative medicine compared to previous times (Albassam et al. 2024). Barberry, a plant in the Berberidaceae family, is widely used in traditional medicine in Iran for neurodegenerative diseases due to its alkaloid compounds, which have antioxidant and anti‐inflammatory properties (Imanshahidi and Hosseinzadeh 2008). It is effective in treating various diseases by reducing the amount of ROS in cells, thus fighting OS (Rad et al. 2022). Research shows that a combination of barberry and DZN increases antioxidants in the cerebellum and cerebrum of rats, unlike DZN alone. In addition, the activity of brain AChE, an enzyme related to brain function, is boosted by barberry extract. This highlights the potential benefits of barberry as a valuable remedy for various ailments (Dini et al. 2024).

Numerous studies have demonstrated that exposure to DZN can lead to not only an elevation in TOS and MDA, as important biomarkers of OS, but also a reduction in TAC, SOD, and glutathione (GSH) levels (Weiss and Deutschman 2014; Z. Singh et al. 2014). Our findings showed that DZN exposure resulted in a significant increase in MDA levels and a decrease in SOD content in the brain tissue of rats. These findings were consistent with similar studies that demonstrated that DZN exposure can lead to a significant increase in MDA levels and a decrease in SOD content. In contrast, published studies reported that barberry extract increased TAC and SOD levels (Hassanpour and Alizadeh 2016), and it induces a significant decrease in both TOS and MDA levels (Emamat et al. 2022). The presence of bioactive compounds such as anthocyanins and BBR in barberry is responsible for these observed effects (Aslam et al. 2013). Our investigation, in alignment with previous research, indicates that the combination of barberry and DZN in rats resulted in a more pronounced increase in TAC levels and a greater decrease in TOS levels compared to rats treated with barberry alone, highlighting the potent effects of this combination.

After biochemical evaluations and confirmation of the role of OS and enzyme inhibition in DZN neurotoxicity, behavioral changes in animals were examined. Our results in the OFT experiment showed that in the groups exposed to DZN, the animals' locomotor activity increased due to an increase in anxiety levels and a decrease in the time spent in the central area of the apparatus. Conversely, co‐treatment with barberry extract led to an improvement in locomotor activity and anxiety levels in the animals. Our findings were consistent with those of Savy et al. (2015) and Roegge et al. (2008). They reported that chronic exposure to DZN can cause changes in the locomotor activity of animals depending on the duration and level of exposure. In order to assess long‐term memory, the *Y*‐maze test was used. The findings of this study showed that DZN exposure caused long‐term memory impairment. Treatment with barberry extract improved this impairment. It can be inferred that barberry possesses numerous therapeutic qualities for addressing DZN‐related disorders. This extract could possibly serve as a significant indicator for minimizing disruptions in the rats’ movement abilities (Slotkin et al. 2007). According to studies, the mechanism of neurobehavioral symptoms following long‐term exposure to DZN is still not clearly understood. A number of studies showed that, depending on the route of exposure and bioavailability, OP compounds enter the blood after absorption. Due to their lipophilicity, they can be distributed to fat‐rich tissues, especially brain tissue. It is likely that, in OP exposure, excess acetylcholine simply disrupts the balance of neurotransmitter systems in cortical and subcortical areas or alters the acetylcholine feedback loop (Papandreou et al. 2009; Baltazar et al. 2014). Another mechanism that may be postulated to explain the neurological effects is attributed to an imbalance between excitatory and inhibitory neurons in different brain regions (Arendt 2009). Histopathological findings obtained from brain tissue indicated that behavioral changes in DZN exposure can be presumably related to damage to the hippocampus. Brain tissue results showed that exposure to DZN can cause enduring changes in the development of neural cells. The administration of DZN led to a significant decrease in the normal neurons found in the pyramidal layer and throughout the hippocampus. This reduction may be attributed to the suppression of enzyme activity (Seo et al. 2013). The presence of cell nuclei and pyknosis signifies the degradation of these cells (Ezabadi et al. 2019). The administration of barberry extract in the group treated with DZN demonstrated its efficacy in enhancing tissue conditions, particularly regarding the morphology and the quantity of normal neurons within the pyramidal layer of the hippocampus.

## 5 Conclusion

The present investigation aims to explored the safeguarding impact of barberry extract on the level of OS and behavioral alterations in male rats' brain tissue affected by DZN poisoning. Our findings indicate that the use of pesticides should be controlled at the community level. Barberry extract demonstrates a strong capacity to combat OS, which is crucial in preventing damage to brain tissues. This is achieved through the reduction of TOS and MDA biomarkers, as well as the enhancement of antioxidant enzyme activity, including SOD, TEC, and GSH. Furthermore, the consumption of barberry has been shown to alleviate behavioral and memory disorders caused by DZN intake. Further research is required to bolster these findings, with a focus on conducting further numerous experimental studies to explore the different facets of the positive impacts of barberry extract in mitigating OS, and consequently, mitigating the adverse effects of DZN. There were some limitations in this study that should be considered in future studies, including DZN administration. Also, it is required to investigate molecular mechanisms involved in the anti‐inflammatory and antioxidant effects of barberry against DZN‐induced brain impairment. Undoubtedly, such studies can help to provide a novel vista in the nature‐based therapeutic strategy for pesticides.

## Author Contributions

**Fereshteh Mehri**: Writing – original draft, Writing – review and editing. **Ahmad Fadai**: writing – review and editing, writing – original draft. **Elham Shiri**: funding acquisition. **Razieh Parvareshi Hamrah**: investigation.

## Funding

This work was supported by the Vice Chancellor for Research and Technology at Hamadan University of Medical Sciences (Grant No. 140302181274).

## Conflicts of Interest

The authors declare no conflicts of interest.

## Data Availability

The authors have nothing to report.
